# Evaluating the Clinical Performance of the Elecsys pTau217 Plasma Immunoassay to Detect Amyloid Pathology in a Routine Clinical Practice Cohort

**DOI:** 10.1002/alz70856_096679

**Published:** 2025-12-24

**Authors:** Sayuri Hortsch, Niels Borlinghaus, Alexander Jethwa, David Caley, Annunziata Di Domenico, Craig Ritchie

**Affiliations:** ^1^ Roche Diagnostics GmbH, Penzberg, Bavaria, Germany; ^2^ Roche Diagnostics Ltd, Burgess Hill, West Sussex, United Kingdom; ^3^ Roche Diagnostics International Ltd, Rotkreuz, Zug, Switzerland; ^4^ Scottish Brain Sciences, Edinburgh, Scotland, United Kingdom; ^5^ University of St Andrews, St Andrews, Scotland, United Kingdom

## Abstract

**Background:**

This study evaluated the clinical performance of the high‐throughput, fully‐automated prototype Elecsys^®^ Phospho‐Tau (217P) Plasma immunoassay (Roche Diagnostics International Ltd, Rotkreuz, Switzerland) to detect amyloid pathology. A cohort reflective of routine clinical practice was used to assess differences in the discriminative ability of phosphorylated tau 217 (pTau217) with respect to amyloid positron emission tomography (PET) visual read status (a regulatory approved reference method) and centiloid classifications (an exploratory method used in research).

**Method:**

In this prospective, multicenter study, EDTA plasma samples were collected from adults aged 55–80 years old with subjective or objective cognitive impairment, under evaluation for Alzheimer's disease or other causes of cognitive decline. Patients with early‐stage disease such as subjective cognitive decline (SCD), mild cognitive impairment (MCI), or mild dementia were included. Patients were recruited at 12 clinical sites in Europe and the United States across primary and secondary care. Plasma pTau217 concentrations were measured using the prototype pTau217 plasma immunoassay at a single site (Roche Diagnostics GmbH). The discriminative ability of pTau217 to detect amyloid pathology with respect to amyloid PET visual read status and binary centiloid classifications (at selected cut‐offs; 24.1–50.0) was assessed using receiver operating characteristic curve analysis.

**Result:**

In total, 603 patients were enrolled who were heterogeneous in terms of sex, race, comorbidities, and disease stages, reflective of routine clinical practice. Across primary and secondary care settings, the prototype pTau217 plasma immunoassay exhibited an area under the curve (AUC) of 0.917 compared with PET visual read and an AUC between 0.896–0.925 when compared with centiloid classifications at various selected cut‐offs. The choice of centiloid cut‐off influenced the observed amyloid positivity prevalence and the sensitivity and specificity of the prototype pTau217 plasma immunoassay.

**Conclusion:**

The prototype pTau217 plasma immunoassay accurately detected amyloid positivity in a real‐world cohort of patients presenting with cognitive decline across primary and secondary care. Together with the full automation and high‐throughput of the assay, these data support potential implementation into routine clinical practice. In future, if performance is assessed with respect to centiloid classifications, the influence of centiloid cut‐offs should be considered.

